# Supplementary material for: Molecular Dynamics Mappings of the CCT/TRiC Complex-Mediated Protein Folding Cycle Using Diffracted X-ray Tracking
Source: Int J Mol Sci. 2023 Oct 3;24(19):14850. doi: 10.3390/ijms241914850 (PMC10573753; doi:10.3390/ijms241914850)
Supplement: Supplementary file 1 [file ijms-24-14850-s001.zip › ijms-2608620-supplementary.pdf]

Supplementary information

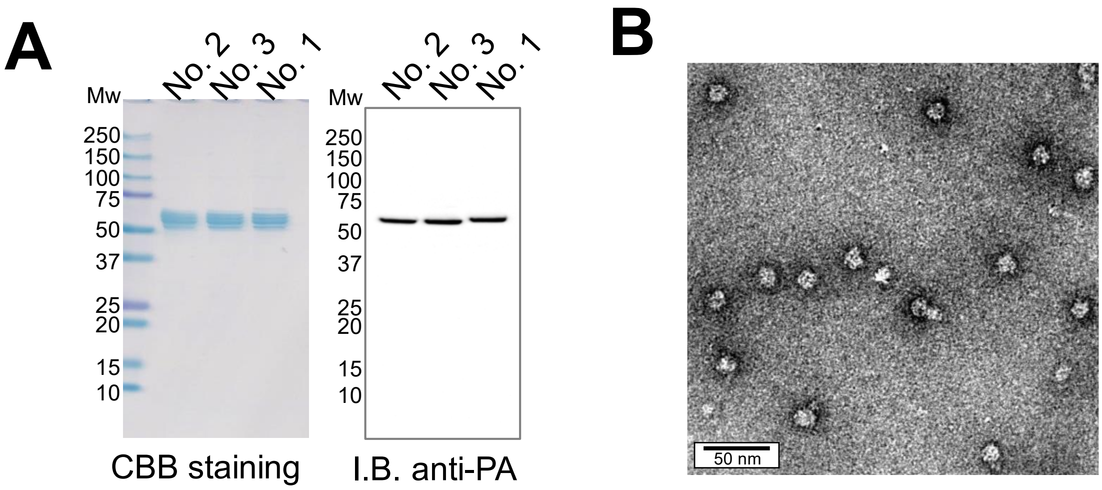

Supplementary Figure S1. Purified CCT Complex Proteins

(A) The purified CCT complex proteins with PA-tag with three different regions (No.1, No.2, No.3) were detected using Coomassie Brilliant Blue (CBB) staining (left) and western blotting analysis with an anti-rabbit monoclonal PA-tag antibody (right). (B) The CCT complex was visualized by negative-stain electron microscopy (EM), with No.2 used as a representative example.

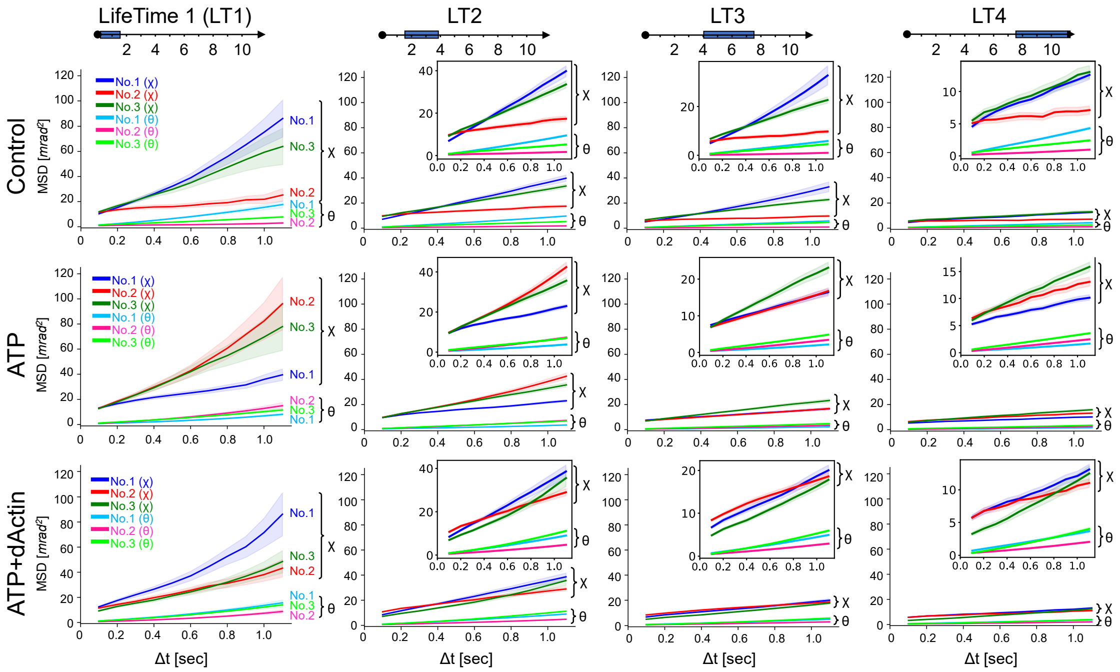

Supplementary Figure S2. Mean Square Displacement (MSD) Curves of the CCT Complex Motion for the χ Axis under Lifetime Classification

From left to right, the MSD curves for LifeTime 1 (LT1), LT2, LT3, and LT4 are shown, and the results for the Control, ATP, and ATP+dActin conditions are shown from top to bottom. The insets on the right above are zoomed-in views of the Y-axis (MSD values) of the graphs for each condition of LT2, LT3, and LT4. The 95% confidence levels are indicated with shading.

Condition		LT1	LT2	LT3	LT4
Control	No.1	4.70 (±0.83)	2.04 (±0.05)	1.73 (±0.17)	0.48 (±0.06)
	No.2	0.74 (±0.20)	0.46 (±0.06)	0.23 (±0.05)	0.12 (±0.04)
	No.3	3.31 (±0.29)	1.48 (±0.04)	1.00 (±0.03)	0.46 (±0.04)
ATP	No.1	1.51 (±0.26)	0.78 (±0.09)	0.55 (±0.01)	0.30 (±0.03)
	No.2	5.07 (±1.06)	2.01 (±0.22)	0.60 (±0.01)	0.42 (±0.04)
	No.3	4.06 (±0.32)	1.60 (±0.05)	1.01 (±0.03)	0.61 (±0.03)
ATP+dActin	No.1	4.35 (±1.14)	1.87 (±0.05)	0.81 (±0.05)	0.44 (±0.03)
	No.2	1.90 (±0.20)	1.11 (±0.07)	0.62 (±0.07)	0.31 (±0.05)
	No.3	2.34 (±0.44)	1.74 (±0.28)	0.79 (±0.05)	0.58 (±0.04)

Supplementary Table S1. Summary of the Angular Diffusion Coefficient in χ directions
After classifying the trajectories into these four lifetime groups (Supplementary Figure 2 and Figure 4), MSD curves were fitted at the time interval of 0.1 second with least-squares fitting to the following equation, $MSD = 4Dt + A$, where MSD is the mean square angular displacement, D (mrad^2/s) is the angular diffusion constant, t is time interval, A is intercept of the MSD curve.
